# Targeting renal cell carcinoma with NVP-BEZ235, a dual PI3K/mTOR inhibitor, in combination with sorafenib

**DOI:** 10.1186/1476-4598-10-90

**Published:** 2011-07-26

**Authors:** Didier Roulin, Laurent Waselle, Anne Dormond-Meuwly, Marc Dufour, Nicolas Demartines, Olivier Dormond

**Affiliations:** 1 Department of Visceral Surgery, Centre Hospitalier Universitaire Vaudois and University of Lausanne, Pavillon 3, Av. de Beaumont, 1011 Lausanne, Switzerland

## Abstract

**Background:**

Targeted therapies for metastatic renal cell carcinoma (RCC), including mammalian target of rapamycin (mTOR) inhibitors and small-molecule multikinase inhibitors, have produced clinical effects. However, most patients acquire resistance over time. Thus, new therapeutic strategies need to be developed. Here, we evaluated the effect of the dual PI3K/mTOR inhibitor NVP-BEZ235, in combination with the multikinase inhibitor sorafenib on renal cancer cell proliferation and survival *in vitro *as well as on tumor growth *in vivo*.

**Methods:**

The renal carcinoma cell lines 786-0 and Caki-1 were treated with NVP-BEZ235 or sorafenib, either alone or in combination. Tumor cell proliferation and apoptosis were investigated *in vitro*. The anticancer efficacy of NVP-BEZ235 alone, or in combination with sorafenib, was also evaluated on RCC xenografts in nude mice.

**Results:**

Treatment of 786-0 and Caki-1 cells with NVP-BEZ235 or sorafenib resulted in reduced tumor cell proliferation and increased tumor cell apoptosis *in vitro*. The combination of NVP-BEZ235 and sorafenib was more effective than each compound alone. Similarly, *in vivo*, NVP-BEZ235 or sorafenib reduced the growth of xenografts generated from 786-0 or Caki-1 cells. The antitumor efficacy of NVP-BEZ235 in combination with sorafenib was superior to NVP-BEZ235 or sorafenib alone.

**Conclusions:**

Our findings indicate that the simultaneous use of NVP-BEZ235 and sorafenib has greater antitumor benefit compared to either drug alone and thus provides a treatment strategy in RCC.

## Introduction

Renal cell carcinoma (RCC) is a highly vascularized tumor which accounts for 3% of all malignancies in adults [1]. Most symptomatic patients present with advanced metastatic disease, which has a poor prognosis. Traditional chemotherapy, hormonal therapy or radiation are not effective in the treatment of advanced RCC, and immunotherapy (including IL-2 and interferon-α) provides only limited benefit [2]. Nevertheless, based on the molecular biology of RCC, new therapeutic strategies have recently emerged in the management of advanced disease. Indeed, a characteristic of RCC is the frequent inactivation of the Von Hippel Lindau protein (pVHL), which occurs in 50 to 60 percent of patients with sporadic RCC [3]. The molecular consequences of pVHL mutations result in the upregulation of Hypoxia-Inducible Factor-1α (HIF-1α) which induces the transcription of hypoxia responsive genes such as Vascular Endothelial Growth Factor (VEGF) [4]. In consequence, loss of pVHL results in VEGF production and induction of angiogenesis.

Encouraging clinical studies show that agents targeting VEGF and tumor angiogenesis significantly prolong progression-free survival in patients with RCC [1,5]. Among those agents, sorafenib has been approved for the treatment of advanced RCC [6]. Initially identified as a Raf kinase inhibitor, sorafenib also blocks the kinase activities of several receptors including VEGF receptor 1, 2, 3 and platelet derived growth factor receptor beta [7]. Sorafenib exhibits antitumor activity in several experimental models of renal cancer, primarily by inhibiting angiogenesis [8].

In addition to sorafenib, allosteric inhibitors of the mammalian target of rapamycin (mTOR) have also been approved for the treatment of advanced RCC. The rationale of targeting mTOR in RCC is related to the observation that mTOR regulates the expression of HIF-1α [9]. Two such inhibitors, temsirolimus [10] and everolimus [11], have significant activity in patients with advanced RCC and prolong the progression-free survival. However, the responses are short lived and most of the patients finally develop resistance [12]. These limited benefits observed in clinical trials are partially explained by experimental evidences where treatment of cells with rapamycin, or its analogs temsirolimus and everolimus, activates the PI3K/Akt signaling pathway by the removal of a negative feedback loop [13]. In turn, the activation of PI3K/Akt results in the activation of proliferative and pro-survival signals that counteract the anticancer efficacy of rapamycin. Furthermore, mTOR exists in two different complexes, mTORC1 and mTORC2. While mTORC1 is sensitive to rapamycin, mTORC2 is not [14]. Finally, not all the functions of mTORC1 are targeted by rapamycin [15]. To overcome these limitations, a new generation of agents targeting the ATP-binding domain of mTOR and inhibiting both mTORC1 and mTORC2 has been developed [16]. Among these agents, NVP-BEZ235 is a dual PI3K/mTOR inhibitor currently in clinical development [17]. The antitumor efficacy of NVP-BEZ235 has been demonstrated in numerous preclinical models [18-20], including RCC where its anticancer efficacy is shown to be superior to rapamycin [21]. Interestingly, NVP-BEZ235 has little effect on tumor angiogenesis in RCC suggesting that its antitumor efficacy may be potentiated in combination with anti-angiogenic therapy [21].

Despite having improved the clinical outcome of patients with RCC, targeted therapies are not associated with long lasting responses. Consequently, there is a strong need to develop new therapeutic strategies for the treatment of RCC. In this report, we have analyzed the effects of NVP-BEZ235 in combination with the anti-angiogenic compound sorafenib on renal cancer cell lines *in vitro *and on renal tumor xenografts *in vivo*.

## Material and Methods

### Cell lines, antibodies and reagents

The human renal cell carcinoma cell lines 786-0 and Caki-1 were obtained from the American Type Culture Collection and cultured in DMEM medium supplemented with 10% fetal bovine serum (FBS) and 1% penicillin-streptomycin. Cells were incubated at 37°C at 5% CO_2_. Antibodies directed against phospho-Akt (S473), Akt, phospho-S6 ribosomal protein (Ser235/236), S6 ribosomal protein, phospho-MAPK (Thr202/Tyr204), MAPK, cleaved caspase-3 and actin were from Cell Signaling. Antibody against CD31 was purchased from BD Biosciences. NVP-BEZ235 and sorafenib were purchased from LC Laboratories.

### Cell count

Cells were plated in six-well plates (Costar) at a density of 100 000 cells/well and cultured in DMEM 10% FBS. Twelve hours later, cells were treated with increasing doses of NVP-BEZ235 (10-1000 nM), sorafenib (10 μM), a combination of both or DMSO as a control for 48 or 72 hours. Subsequently, adherent cells were collected and trypan-blue negative cells were counted using a Neubauer hemocytometer.

### MTS proliferation assay

Caki-1 or 786-0 cells were plated on 96-well plates (Costar) at 10'000 cells per well and cultured in DMEM 10% FBS. Twelve hours later, cells were treated with NVP-BEZ235 1 μM, sorafenib 10 μM, a combination of both or DMSO as a control. Cellular proliferation was monitored after 48 or 72 hours of treatment with the CellTiter 96^® ^AQ_ueous _One Solution (Promega Corporation) colorimetric assay by following the manufacturer's instructions. The MTS compound is reduced by living cells into a formazan product whose quantity is directly proportional to the number of cells in culture. The quantity of formazan product is measured by the amount of 490 nm absorbance.

### BrdU incorporation assay

Cells were plated on coverslips and treated with the indicated inhibitor for 24 hours. 5-bromo-2'-deoxyuridine (BrdU) at a final concentration of 10 μM was added to the culture medium for the last 12 hours. Subsequently, cells were fixed with paraformaldheyde (4%) for 10 min, washed twice with PBS and incubated with HCl 2 N for 2 min. Cells were extensively washed in PBS and immunocytofluorescence was done with mouse anti BrdU antibody (DAKO), and the fluorochrome conjugated secondary antibody against mouse Ig (alexa 594, Invitrogen). The nuclei were counterstained with DAPI. Immunostained cells were observed under epifluorescent microscope IX81 (Olympus). BrdU and DAPI positive cells were counted using a computer-assisted image analysis station (Mercator, Explora Nova). Results were expressed as the ratio of BrdU- to DAPI-positive cells.

### Apoptosis Assay

The Cell Death Detection ELISA^plus ^kit (Roche) was used to measure apoptosis. Caki-1 and 786-0 cells were seeded in 96-well plates at 30,000 cells per well and grown in serum-free medium at 37°C. Twelve hours later, cells were treated with NVP-BEZ235 (1 μM), sorafenib (10 μM), a combination of both, or DMSO as a control, for 24 hours. Subsequently cells were harvested and apoptosis was determined following the manufacturer's instructions. Results are represented as the mean enrichment factor (absorbance of the treated cells/absorbance of the control cells).

### Cell cycle analysis

Caki-1 and 786-0 cells were treated with NVP-BEZ235 (1 μM), sorafenib (10 μM), a combination of both, or DMSO as a control for 48 hours. Cells were collected and processed for FACS analysis as previously described [22].

### Western Blot Analysis

Western Blot analysis were performed as previously described [22].

### Xenograft model

Animal experiments were in accordance with the Swiss federal animal regulations and approved by the local veterinary office. Female nude eight-week old mice were purchased from Charles River Laboratories. Caki-1 or 786-0 cells at 3 × 10^6 ^were injected subcutaneously into the flank. Once the tumor xenografts reached 25 mm^3 ^mice were randomized into different groups (n = 5/group) and treated once daily by gavage with vehicle, Sorafenib (15 mg/kg/day), NVP-BEZ235 (30 mg/kg/day), or in combination. NVP-BEZ235 was solubilized in one volume of N-methylpyrrolidone and further diluted in nine volumes of PEG 300. Sorafenib was dissolved in Cremophor EL/ethanol (Sigma) at 4-fold (4×) and further diluted to 1× with water. Tumor volumes were measured using caliper measurements every day and calculated with the formula V = π/(6a^2^b) where *a *is the short axis and *b *the long axis of the tumor. Animals were sacrificed after 20 days of treatment and the tumors were excised and weighed.

### Immunochemistry

Tumor xenografts were carefully removed and rapidly frozen in OCT compound (Tissue-Teck) on dry ice. Ten μm transverse sections were cut on a cryostat (CM 1850, Leica), and processed for immunolabeling with an anti-CD31 antibody (1:20, MEC13.3, BD Biosciences) as previously described [23]. Vessels were manually counted in five high-power fields (HPF) in each tumor. In addition, immunolabeling with an anti-Ki-67 (Novocastra) antibody was also performed as described by others [24].

### Statistical analysis

Comparisons between groups were done using one-way ANOVA followed by Dunnett's post-hoc test. Comparisons between groups for tumor volume progression were done using repeated measures ANOVA. All calculations were done using IBM SPSS Statistics 18. Values of p < 0.05 were considered statistically significant.

## Results

### Antitumor activity of NVP-BEZ235 alone or in combination with sorafenib on 786-0 and Caki-1 cells in vitro

**Figure 1:**
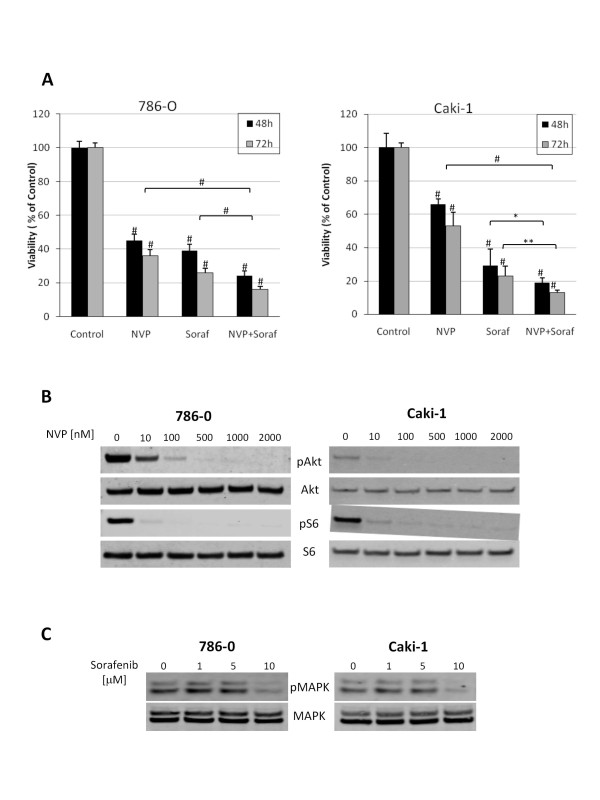
**NVP-BEZ235 and Sorafenib inhibit the growth of renal cancer cell lines**. A, 786-0 and Caki-1 cellular growth was monitored with colorimetric MTS assay after 48 hours of treatment with NVP-BEZ235 (NVP, 1 μM) or Sorafenib (Soraf, 10 μM) either alone or in combination, or DMSO (Control). Columns, mean cell viability relative to control of four independent experiments; bars, SD. *, P < 0.05, **, P < 0.01, #, P < 0.001 compared to control, or otherwise as specified by brackets. B, NVP-BEZ235 inhibits PI3K/mTOR pathway in renal cancer cells. 786-0 and Caki-1 cells were treated with increasing doses of NVP-BEZ235 or DMSO (Control) for 4 hours. Cells were subsequently lysed and lysates were examined for phospho-Akt (Ser 473), Akt, phospho-S6 (Ser 235/236) or S6 expression level by Western blot analysis. C, 786-0 and Caki-1 cells were treated with increasing doses of sorafenib or DMSO as a control for 4 hours. Cells were processed as under panel B and analyzed for phospho-MAPK and MAPK (Thr202/Tyr204). The illustrated blots are representative of three similar experiments.

To evaluate the efficacy of combined NVP-BEZ235 and sorafenib treatment on renal cancer cell, 786-0 and Caki-1 cells were exposed to NVP-BEZ235 and sorafenib either alone or in combination for 48 and 72 hours and analyzed by MTS assay. Growth of 786-0 and Caki-1 cells was significantly inhibited by each drug alone (Figure 1A). The combination of both drugs further significantly decreased renal cancer cell growth compared to single drug treatment. NVP-BEZ235 was used at a concentration of 1 μM which proved to be efficient in inhibiting mTORC1 and mTORC2 as assessed by the inhibition of the phosphorylation of S6 ribosomal protein and Akt, downstream effectors of mTORC1 and mTORC2 respectively (Figure 1B). Similarly, cells were exposed to 10 μM of sorafenib, a concentration at which sorafenib reduced Raf kinase activity as observed by the reduction of MAPK phosphorylation (Figure 1C).

### Effect of NVP-BEZ235 alone or in combination with sorafenib on renal cancer cell proliferation

**Figure 2:**
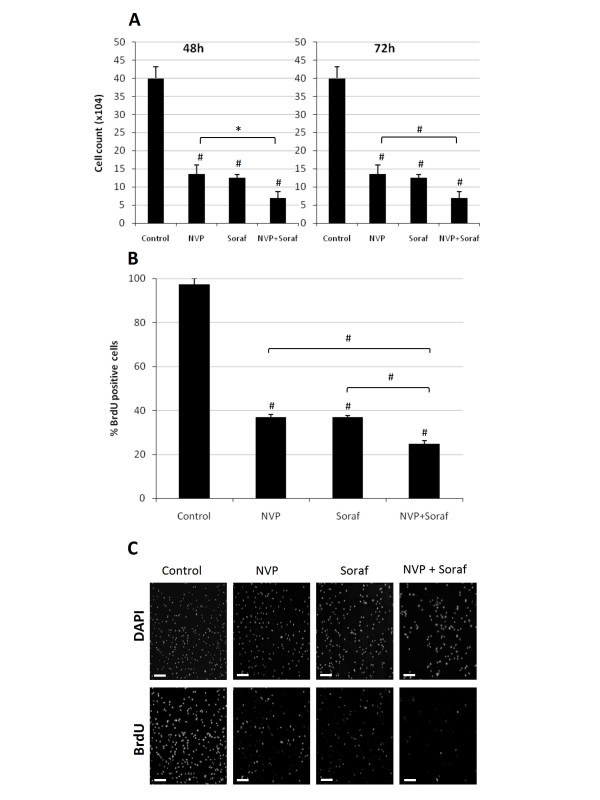
**Effect of NVP-BEZ235 alone or combined with Sorafenib on renal cancer cell proliferation**. A, 786-0 cells were treated with NVP-BEZ235 (NVP; 1 μM) alone or combined to Sorafenib (Soraf; 10 μM). Cells were counted after 48 or 72 hours of treatment as described under Materials and Methods. Columns, mean cell count of three independent experiments; bars, SD. B, BrdU uptake of 786-0 cells treated with NVP-BEZ235 (NVP, 1 μM) or Sorafenib (Soraf, 10 μM) either alone or in combination, or DMSO (Control), for 24 hours. Results are quantified as percentage of positive 786-0 cells for BrdU incorporation. Columns, mean percentage of three independent experiments; bars, SD. C, samples processed and analyzed under a fluorescence microscope. (Magnification 100×. Scale bar 100 μm). *, P < 0.05, #, P < 0.001 compared to control, or otherwise as specified by brackets.

We next performed proliferation assays to determine whether the reduction in cell growth observed with NVP-BEZ235 and sorafenib (Figure 1A) was due to a reduction in cell proliferation. 786-0 cells were exposed to NVP-BEZ235 or sorafenib, alone or in combination and cell number was determined after 48 or 72 hours of treatment. We observed that NVP-BEZ235 as well as sorafenib significantly reduced 786-0 cell number after 48 and 72 hours compared to untreated cells (Figure 2A). Similarly, BrdU incorporation was more significantly reduced in cells treated simultaneously with NVP-BEZ235 and sorafenib compared to cells treated with NVP-BEZ235 or sorafenib alone (Figure 2B and 2C). Similar results were obtained with Caki-1 cells (data not shown). Collectively these results suggest that the antiproliferative efficacy of NVP-BEZ235 or sorafenib on renal cancer cell is significantly improved when both drugs are used simultaneously.

### Effect of NVP-BEZ235 alone or in combination with sorafenib on renal cancer cell apoptosis

**Figure 3:**
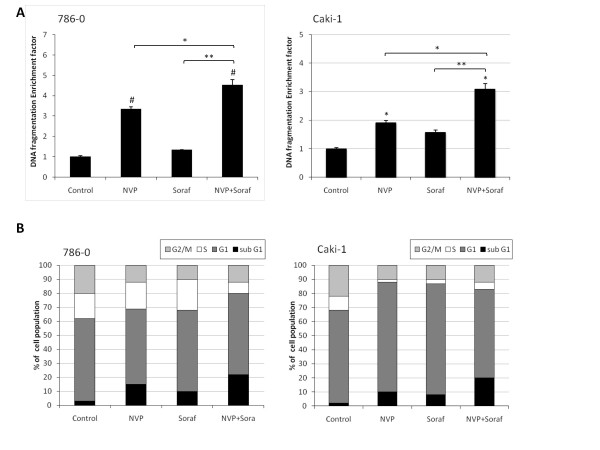
**Effect of NVP-BEZ235 alone or combined with Sorafenib on renal cancer apoptosis**. A, 786-0 (left panel) or Caki-1 cells (right panel) were treated for 24 hours with NVP-BEZ235 (NVP, 1 μM) or Sorafenib (Soraf, 10 μM) either alone or in combination, or DMSO (Control). Cells were harvested and apoptosis was measured by quantifying DNA fragmentation. Columns, mean enrichment factor at 405 nm of three independent experiments; bars, SD. B, 786-0 and Caki-1 cells were treated as in A for 48 hours and processed for cell cycle analysis. One of three similar experiments is shown. *, P < 0.05, **, P < 0.01, #, P < 0.001 compared to control, or otherwise as specified by brackets.

We further analyzed the potential of NVP-BEZ235 alone or in combination with sorafenib to induce renal cancer cell apoptosis. 786-0 and Caki-1 cells were treated with NVP-BEZ235, sorafenib or a combination of both and cell apoptosis was determined after 24 hours of treatment using a cell death detection ELISA. NVP-BEZ235 and to a lesser extend sorafenib induced apoptosis as reflected by an increased DNA fragmentation in 786-0 and Caki-1 cells. This pro-apoptotic effect was also potentiated when both drugs were used in combination compared to single therapy (Figure 3A). Consistent with this finding, we also found by cell cycle analysis that combined therapy resulted in a more prominent sub-G1 population when compared to monotherapy (Figure 3B). Taken together these results show that the pro-apoptotic effect of NVP-BEZ235 in combination with sorafenib is superior to single treatment.

### Effect of NVP-BEZ235 alone or in combination with sorafenib on the growth of renal cancer xenografts

**Figure 4:**
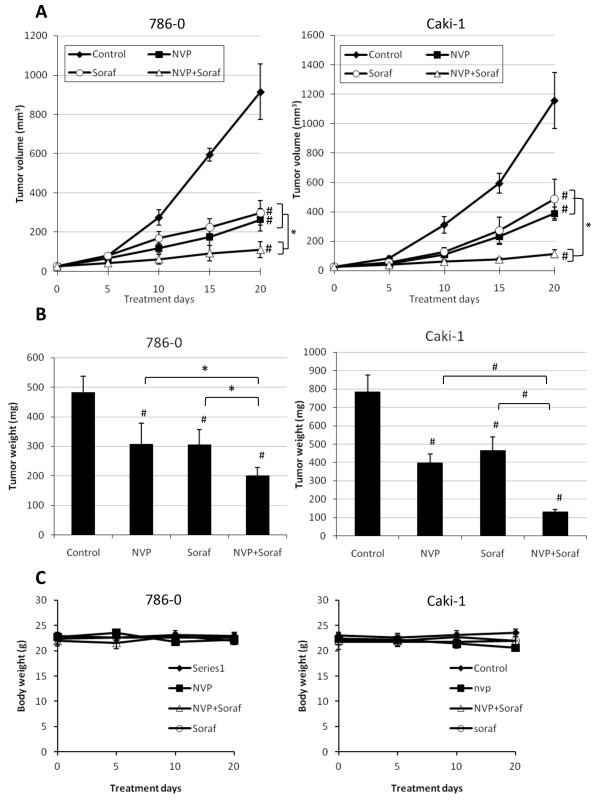
**Effect of NVP-BEZ235 alone or combined with Sorafenib on the growth of 786-0 and Caki-1 xenografts**. A, An equal amount of 786-0 (left panel) or Caki-1 (right panel) cells were harvested and injected subcutaneously into nude mice. Once the tumor reached 25 mm^3 ^mice were randomized into four groups and treatments were started with vehicle (Control), NVP-BEZ235 (NVP, 30 mg/kg/day po), Sorafenib (Soraf, 15 mg/kg/day po), or a combination of both (NVP+Soraf). Five mice were included in each group. Tumor volumes were evaluated using caliper measurements and calculated with the formula V = π/6 × *a*^2 ^× *b *where *a *is the short axis and *b *the long axis of the tumor. Points, mean value of tumor volume; bars, SD. B, After 20 days of treatment, mice were sacrificed, tumor xenografts were harvested and tumor weight was measured. Columns, mean tumor weight (five tumor xenografts in each group); bars, SD. *, P < 0.05, #, P < 0.001 compared to control, or otherwise as specified by brackets. C, Body weights of mice were measured to assess toxicity of the treatments. Points, mean body weight (five mice in each groups); bars, SD.

We next studied the effect of NVP-BEZ235 alone or in combination with sorafenib on the growth of 786-0 and Caki-1 xenografts. Nude mice bearing 786-0 or Caki-1 tumor xenografts were treated with NVP-BEZ235, sorafenib or a combination of both drugs for 20 days. We used low doses of NVP-BEZ235 (30 mg/kg/day) since we observed in preliminary studies that these were sufficient to block mTORC1 and mTORC2 in tumor xenografts (data not shown). In addition, we used 15 mg/kg/day of sorafenib which has been previously shown to reduce the growth of renal cancer xenografts [8]. The tumor size (Figure 4A) and weight (Figure 4B) of NVP-BEZ235- or sorafenib-treated xenografts were significantly smaller in comparison with untreated (control) xenografts. Moreover, the growth of combined NVP-BEZ235 and sorafenib treated xenografts was significantly reduced when compared to monotherapy. Overall, the treatments were tolerated without evident toxicity. All animals survived after 20 days of treatment and no significant body weight loss was observed (Figure 4C). Taken together, these results show that the anti-cancer efficacy of NVP-BEZ235 combined with sorafenib is greater than either drug used alone.

### Effect of NVP-BEZ235 alone or in combination with sorafenib on tumor cell proliferation and survival and tumor angiogenesis

**Figure 5:**
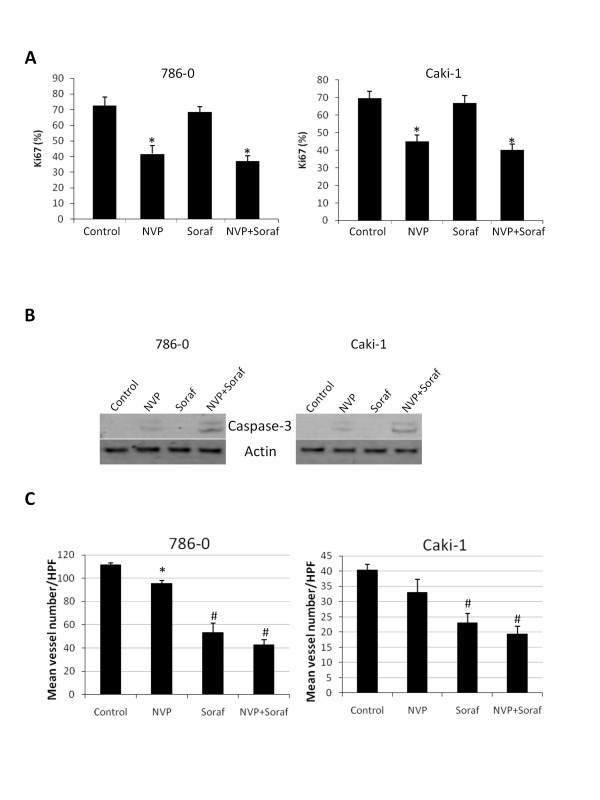
**Effect of NVP-BEZ235 (NVP) alone or in combination with Sorafenib (Soraf) on tumor cell proliferation, apoptosis and tumor angiogenesis**. A, Frozen sections of tumor xenografts treated as in Figure 4 and harvested after 20 days of treatment were stained with an anti-Ki-67 antibody. The effect of the different treatments on Ki-67 positivity was quantified and expressed as % of cells positive for Ki-67/total number of cells (300 cells counted per tumor; five tumors in each group). Columns, mean % of cells positive for Ki-67 staining; bars, SD. B, Tumor lysates were generated from xenografts and analyzed for cleaved caspase-3 and actin expression. C, Frozen sections of tumor xenografts were stained with an anti-CD31 antibody. Vessels were manually counted in five high-power fields (HPF) in each tumor. Columns, mean vessel number per HPF (three tumor xenografts analyzed in each group); bars, SD. *, P < 0.05, #, P < 0.001 compared to control.

To better understand the mechanism of action of NVP-BEZ235 and sorafenib in vivo, tumor xenografts were harvested after 20 days of treatment and processed for various analysis. Immunostainings of Ki-67 and CD31 were used to determine tumor cell proliferation and angiogenesis respectively. Western Blot analysis of tumor xenografts for cleaved caspase-3 expression was used to detect cell apoptosis. NVP-BEZ235 reduced cell proliferation and induced apoptosis in both 786-0 and Caki-1 tumor xenografts (Figure 5A and 5B). NVP-BEZ235 slightly decreased tumor vasculature which was only significant in 786-0 xenografts (Figure 5C). Sorafenib had no effect on tumor cell proliferation and did not induce cleaved caspase-3 expression. However, sorafenib significantly reduced tumor angiogenesis. Combining NVP-BEZ235 and sorafenib had no additive effects on tumor cell proliferation and tumor angiogenesis. In contrast, cleaved caspase-3 expression was increased when mice were treated concomitantly with NVP-BEZ235 and sorafenib compared to NVP-BEZ235 alone. Taken together these results suggest that, in 786-0 and Caki-1 tumor xenografts, sorafenib potentiates the pro-apoptotic efficacy of NVP-BEZ235.

### Effect of treatment interruption on tumor growth

**Figure 6:**
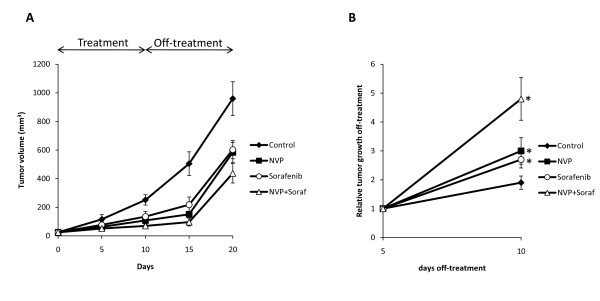
**Effect of treatment cessation on tumor growth**. A, An equal amount of 786-0 cells were injected subcutaneously into nude mice. Once the tumor reached 25 mm^3 ^mice were randomized into four groups and treatments were started with vehicle (Control), NVP-BEZ235 (NVP, 30 mg/kg/day po), Sorafenib (Soraf, 15 mg/kg/day po), or a combination of both (NVP+Soraf). After 10 days, treatments were stopped and tumor growth was further monitored. Points, mean value of tumor volume; bars, SD. Five mice were analyzed in each group. B, Relative tumor volumes (normalized to their size at day 5 off-treatment) at day 10 off-treatment. *, P < 0.05 compared to control.

To next determine the effect on tumor growth induced by the discontinuation of drug administration, nude mice bearing 786-0 cell xenografts were treated with NVP-BEZ235, sorafenib or a combination of both for 10 days. At day 10, drug administration was stopped and tumor growth was monitored for an additional 10 days. We observed that the growth of 760-0 tumor xenografts was still reduced five days after drug interruption, probably reflecting residual inhibition. However, tumors significantly started to grow after 5 days without treatment (Figure 6A). The relative tumor growth was also significantly increased in treated mice compared to untreated mice. The relative tumor growth was further augmented when mice were treated simultaneously with NVP-BEZ235 and sorafenib (Figure 6B).

## Discussion

In this study, we described the antitumor activity of NVP-BEZ235 in combination with sorafenib in renal cancer cells. *In vitro*, the antiproliferative and the pro-apoptotic efficacy of NVP-BEZ235 and sorafenib was significantly increased when both drugs were used in combination compared to monotherapy. Similarly, *in vivo*, the inhibition of tumor growth was greater when both drugs were applied simultaneously compared to either drug alone.

Targeted therapies, including sorafenib, sunitinib, bevacizumab, and mTOR inhibitors, have revolutionized the treatment of metastatic RCC [1]. However, none of these therapies induce complete responses and most of the patients ultimately progress during therapy [12]. Therefore, new strategies are needed to achieve complete responses and block the onset of refractory disease. As it has become evident that most tumors can escape from the inhibition of a single agent, the combination of different targeted agents represent a promising approach [25,26]. Our study showed that combining NVP-BEZ235, a dual PI3K/mTOR inhibitor, and sorafenib might represent a therapeutic strategy in advanced RCC. Consistent with our finding, experimental studies have already shown that combining allosteric inhibitors of mTOR such as rapamycin with sorafenib increases the antitumor effect of both drugs [27]. Clinical trials are currently evaluating the efficacy of this treatment regimen in advanced RCC. Our study further shows that, despite being more potent than rapamycin, the antitumor efficacy of NVP-BEZ235 can also be potentiated in combination with sorafenib.

The mechanism of action of sorafenib has been partially characterized. Since sorafenib is a multi-kinase inhibitor that blocks several targets including VEGFR-1,-2,-3, PDGFRβ and Raf kinases, the molecular mechanisms involved in the antitumor activity of sorafenib might be complex. In our *in vitro *experiments, we observed that sorafenib at 10 μM reduced the phosphorylation of MAPK suggesting that it acts as a Raf kinase inhibitor. In addition, we also found that sorafenib potentiated the anti-proliferative and pro-apoptotic efficacy of NVP-BEZ235 which targets PI3K/Akt/mTOR signaling pathway. Consistent with this observation, previous studies have shown that the antitumor activity of mTOR inhibitors is increased when the Raf/MAPK signaling pathway is concomitantly inhibited [28]. *In vivo*, sorafenib did not reduce cancer cell proliferation and did not induce cancer cell apoptosis. We rather observed that sorafenib reduced tumor angiogenesis suggesting that the mechanism of action of sorafenib is different *in vitro *and *in vivo*.

The rationale to use NVP-BEZ235 with agents targeting angiogenesis is also based on the observation that NVP-BEZ235 has little effect on tumor angiogenesis in xenograft models of RCC. Targeting the PI3K/Akt signaling pathway provides opposite effects on angiogenesis depending on the model used. On one hand, blocking endothelial Akt with rapamycin results in reduced angiogenesis and NVP-BEZ235 decreases VEGF-induced angiogenesis [29-31]. On the other hand, tumors implanted into transgenic mice lacking Akt grow faster and present an increased vasculature [32]. Therefore the angiogenic effect of the inhibition of the PI3K/Akt signaling pathway in endothelial cells may be unpredictable. In this study, we found that NVP-BEZ235 only slightly reduced tumor angiogenesis in 786-0 xenografts. A similar effect was observed in Caki-1 xenografts which was, however, not significant. Consistently, no reduction of tumor angiogenesis was found in RCC xenografts treated with NVP-BEZ235 [21]. Furthermore, an increase of tumor angiogenesis has been described in 786-0 xenografts treated with LY294002, a PI3K inhibitor [33]. Therefore, agents that target the PI3K/Akt pathway have little effect on tumor angiogenesis in renal cancer xenograft models. This suggests that their antitumor efficacy might be increased in combination with anti-angiogenic drugs.

Different options of combination therapy exist, including the inhibition of different targets in the same pathway (vertical blockade), or the inhibition of two separate pathways (horizontal blockade) [26]. As NVP-BEZ235 inhibits multiple effectors in the PI3K/Akt/mTOR signaling pathway, a simultaneous vertical and horizontal blockade is achieved by combining NVP-BEZ235 and sorafenib. The potential problem of such combination therapy is the increased toxicity. Although we did not find any evident toxicity, further studies are required to fully characterize the toxicity profile of this treatment. In particular, side effects should be monitored over a longer period of time.

It was previously reported that NVP-BEZ235 failed to induce renal cancer cell apoptosis in vitro [21]. However, we found here that treatment of 786-0 and Caki-1 cells with NVP-BEZ235 resulted in cell apoptosis as observed by ELISA assay and FACS analysis. In contrast to Cho et al, we performed our apoptotic experiments in the absence of serum which could explain the contradictory results. In fact, we also found that in presence of serum NVP-BEZ235 failed to induce apoptosis of 786-0 and Caki-1 cells (data not shown).

RCC is often associated with a loss of function of pVHL. Previous reports showed that loss of pVHL sensitized renal cancer cells to allosteric inhibitors of mTOR [34]. In this report, we found that NVP-BEZ235 inhibited the growth of VHL -/- 786-0 as well as VHL+/+ Caki-1 cells both in vitro and in vivo, suggesting that NVP-BEZ235 blocks the growth of renal cancer cells regardless of their VHL status. In addition, we also observed that combining NVP-BEZ235 with sorafenib resulted in increased antitumor effects in both cell lines supporting the hypothesis that this therapeutic approach may be effective independently of pVHL status.

## Conclusions

In summary, we reported that the anticancer efficacy of NVP-BEZ235 is potentiated by sorafenib in the context of RCC. Indeed, combining NVP-BEZ235 with sorafenib showed increased antitumor efficacy compared to either drug alone in renal cancer xenografts. Combination treatment also lead to enhanced apoptosis and reduction of renal cancer cell proliferation compared to single therapy. Our results therefore provide a novel treatment strategy in RCC that could be used for the design of clinical studies.

## List of abbreviations

HIF-α: hypoxia-inducible factor-α; mTOR: mammalian target of rapamycin; pVHL: von Hippel Lindau protein; RCC: renal cell carcinoma; VEGF: vascular endothelial growth factor.

## Conflict of interest

The authors declare that they have no competing interests.

## Authors' contributions

DR, LW, ADM and MD performed the experiments and interpreted the experimental findings. DR and OD conceived the study. DR drafted the manuscript. ND and OD wrote the final version of the manuscript. All authors read and approved the final manuscript.
